# Genome instability in blood cells of a *BRCA1*+ breast cancer family

**DOI:** 10.1186/1471-2407-14-342

**Published:** 2014-05-19

**Authors:** Fengxia Xiao, Yeong C Kim, Carrie Snyder, Hongxiu Wen, Pei Xian Chen, Jiangtao Luo, Dina Becirovic, Bradley Downs, Kenneth H Cowan, Henry Lynch, San Ming Wang

**Affiliations:** 1 Department of Genetics, Cell Biology and Anatomy, Omaha, NE 68198, USA; 2 Hereditary Cancer Center, Department of Preventive Medicine, Creighton University, Omaha, NE 68198, USA; 3 Department of Medicine, College of Medicine, Omaha, NE 68178, USA; 4 Department of Biostatistics, College of Public Health, Omaha, NE 68178, USA; 5 Fred & Pamela Buffett Cancer Center, University of Nebraska Medical Center, Omaha, NE 68178, USA

**Keywords:** *BRCA1*+, Familial breast cancer, Exome sequencing, Bioinformatics, Germline mutation, Genome instability

## Abstract

**Background:**

BRCA1 plays an essential role in maintaining genome stability. Inherited *BRCA1* germline mutation (*BRCA1*+) is a determined genetic predisposition leading to high risk of breast cancer. While *BRCA1*+ induces breast cancer by causing genome instability, most of the knowledge is known about somatic genome instability in breast cancer cells but not germline genome instability.

**Methods:**

Using the exome-sequencing method, we analyzed the genomes of blood cells in a typical *BRCA1*+ breast cancer family with an exon 13-duplicated founder mutation, including six breast cancer-affected and two breast cancer unaffected members.

**Results:**

We identified 23 deleterious mutations in the breast cancer-affected family members, which are absent in the unaffected members. Multiple mutations damaged functionally important and breast cancer-related genes, including transcriptional factor *BPTF and FOXP1*, ubiquitin ligase *CUL4B*, phosphorylase kinase *PHKG2*, and nuclear receptor activator *SRA1*. Analysis of the mutations between the mothers and daughters shows that most mutations were germline mutation inherited from the ancestor(s) while only a few were somatic mutation generated *de novo*.

**Conclusion:**

Our study indicates that *BRCA1*+ can cause genome instability with both germline and somatic mutations in non-breast cells.

## Background

BRCA1 maintains genome stability through repairing double-strand DNA damage and other mechanisms [1]. The *BRCA1* germline mutation (*BRCA1*+) is a well-known genetic predisposition for inherited breast cancer [2-4]. Women who inherited *BRCA1*+ have a 60-80% risk of developing breast cancer by the age of 70 [5]. It is believed that *BRCA1*+ leads to breast cancer by causing genome instability [6,7]. Indeed, many efforts have been made to determine the nature of *BRCA1*+ induced genome instability. Cytogenetic studies in *BRCA1*+ familial breast cancer showed the losses of 2q, 4p, 4q, 5q, and 12q [8]; analysis of a breast cancer-derived cell line HCC1937 showed aneuploidy, loss of p53 and PTEN, and loss of heterozygosity (LOH) at multiple loci [9]; analysis in *BRCA1+* basal-like breast cancer identified the losses of the regions containing *RB1, INPP4B, RAD17, RAD50*, and *RAP80*[10], and large-scale chromosomal breakage, copy number loss and LOH [11,12]. Of the three distinct classes of “simple”, “amplifier” and “complex” DNA copy-number alterations defined for breast cancer, *BRCA1*+ breast cancer fits within the “complex” class [13]. Conditional *Brca1* knockout in the mouse model results in breast tumor formation after a long latency, changes in the centrosomes, chromosomal gain and loss in specific segments orthologous to the genetic loci mutated in human breast cancer [14-16]. Data from these studies indicate that many types of genetic defects in the genome are caused by BRCA1+ induced genome instability.

A fundamental question remains to be answered, that is, whether *BRCA1*+ could cause germline genome instability. Most *BRCA1* mutations are founder mutations originated from the ancestor of the affected family passing through multiple generations. While *BRCA1*+ is inherited at the beginning of fertilization of the *BRCA1*+ carriers, breast cancer will only develop in the reproductive age. Between fertilization and reproductive age, are there any genetic changes in *BRCA1*+ carrier genome besides the cancer-targeted breast cell genome, considering the essential roles of BRCA1 in maintaining genome stability? Nearly all genome instability studies in human breast cancer have been focused on breast cancer tissue where germline changes are considered as normal genome variations and disregarded. Therefore, the information derived from previous studies reflects mainly the somatic genome instability in breast cancer cells.

We hypothesize that the loss of BRCA1 function could cause genome instability in non-breast cancer cells. In this study, we used exome sequencing method to analyze the entire coding genes in the genomes of blood cells in a typical *BRCA1*+ breast cancer family. Our study identified multiple recurrent germline and somatic mutations in the genomes of blood cells, highlighting that *BRCA1*+ can cause genome instability in both breast cancer cells and non-breast cancer cells.

## Methods

### The family used for the study

**Figure 1:**
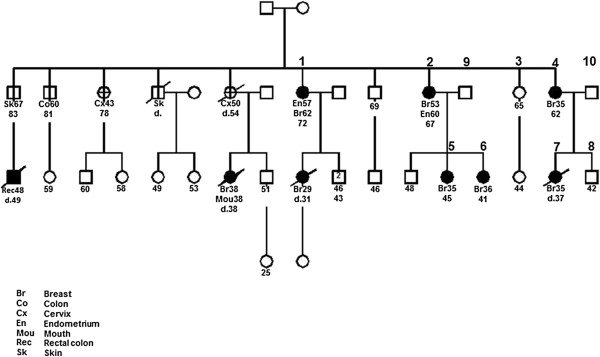
**Pedigree of the *BRCA1*+ family used in the study.** Eight members of this family were sequenced by exome sequencing, of which #1 (62y), #2 (53y), #4 (35y), #5 (35y), #6 (36y), #7 (35y) are breast cancer-affected and #3 (65y) and #8 (45y) are breast cancer-unaffected members. All six affected members are *BRCA1+* whereas the two unaffected members are not*.* #9 and #10 were used in validation to remove the variants transmitted from them to their daughters.

The breast cancer family used in this study contains a heterozygous founder mutation, a 6 kb frameshift duplication comprising exon 13 of *BRCA1* (ins 6 kb exon13-ter1460) that originated from northern British ancestors [17,18]. This mutation is regarded as one of the frequent founder mutations for *BRCA1* mutation testing. Eight family members across two generations were selected for exome sequencing analysis. Of those, six females were diagnosed with breast cancer at the age of 62 (#1), 53 (#2), 35 (#4), 35 (#5), 36 (#6), and 35 years old (#7), and each had inherited the founder mutation. Two members were unaffected at the age of 65 (#3, female) and 45 (#8, male), neither inherited the mutation. #9 and #10 (fathers) were used to remove the variants transmitted to their daughters #5, #6 and #7, accordingly (Figure 1). The use of the samples for the study was approved by the Institutional Review Boards of Creighton University and University of Nebraska Medical Center. All subjects signed the consent form to participate in cancer genetic study and to publish the details.

### Exome sequencing, mapping, variant calling and validation

Genomic DNA from blood cells of the selected individuals was used for this study. Exome library preparation, capture, and sequencing were performed following the Illumina exome sequencing procedures. NimbleGen SeqCap EZ human exome V2.0 kit was used for exome capture. Paired-end sequences (2x100) were collected in the Illumina HiSeq2000 sequencer. The exome data were deposited in NCBI (Accession number SRR949927).

The exome sequences were mapped to the human genome reference sequence hg19 by Bowtie2 using the default parameters in paired mode [19]. The resulting SAM files were converted to BAM files and the duplicates were removed using Picard (http://picard.sourceforge.net). The mapped reads were locally realigned using GATK RealignerTargetCreator. The base quality scores were recalibrated with BaseRecalibrator using dbSNP137 in the GATK resource bundles for hg19.

VarScan 2 [20] and GATK [21] were used for variant calling following the instructions. For VarScan 2, pileup data were generated from BAM files using Samtools [22] mpileup command (with –B parameter to disable BAQ computation), and the default parameters were used with the minimum read depth at 10, minimum base quality at 30; for GATK, UnifiedGenotyper was used for variant calling. BAM files were used for variant calling with GATK v4, release 2.0 with default parameter settings, including stand_call_conf = 30 and stand_emit_conf = 30, the minimum base quality score increased from 17 to 30 using dbSNP137. The variants called by VarScan 2 and GATK were annotated with ANNOVAR using the software provided databases of RefSeq, dbSNP137, 1000 Genomes [23] and ESP6500 from NHLBI Exome Sequencing Project (NHLBI GO Exome Sequencing Project, http://evs.gs.washington.edu/EVS/). The called variants were divided into known variants and novel variants. The known variants were further classified, based on their minor allele frequency (MAF) distribution in ESP6500 or 1000 Genome (≤0.001, and > 0.001). Those with MAF > 0.001 were removed as common normal variants. Those with MAF ≤ 0.001 and novel variants were further classified into synonymous, nonsynonymous, splicing change, stop gain, and stop loss. For the nonsynonymous variants, PolyPhen-2 [24] and SIFT [25] programs were used to identify those with predicted deleterious effects as defined by PolyPhen-2 score [Probably damaging, 0.909-1, Possibly damaging 0.447 - 0.908, Benign 0–0.446 (HumVar score)], and SIFT score covered by ANNOVAR LJB2 (Damaging < 0.05, Tolerant ≥ 0.05) [26]. The final variants include the novel variants and the rare variants (MAF ≤ 0.001) with deleterious effect, splicing alteration, and stop gain/loss. The fragile sites used for the analysis were based on the reference [27].

### Validation

Sanger sequencing was used to validate the variants called by mapping analysis. Sense and antisense primers were designed for each candidate by Primer3 (http://frodo.wi.mit.edu/primer3/). PCR was performed with the same DNA used in exome sequencing (20 ng/reaction), sense and antisense primers (10 pmol), and Taq polymerase (1.25 unit, Promega) at the conditions of denaturing at 95°C 7 minutes, 38 cycles at 95°C 30 seconds, 56°C 30 seconds, 72°C 30 seconds, final extension at 72°C 7 minutes. The amplified DNA products were subject to Big-Dye sequencing reactions. Sequences were collected in a ABI3730 sequencer, and examined by using CLC Genomics Workbench 6.5 program (CLCbio, Cambridge, Massachusetts, USA) to validate the called variants.

## Results

### Exome sequencing and variant calling

We collected paired-end (2x100) exome sequences at 119x coverage on average for each member. We used the following steps for sequence mapping and variant call. 1) Sequences were processed and variants were called by both VarScan 2 and GATK; 2) The variants shared between the affected members and the unaffected members were removed; 3) Nonsynonymous variants were identified; 4) Deleterious variants leading to loss-of-function of the affected genes were predicted by either PolyPhen-2 [24] or SIFT [25] or both programs. 5) The variants shared between the father (#9, #10) and their daughters (#5, #6, #7) were removed upon Sanger sequencing validation; 6) All the remaining variants were validated by Sanger sequencing to confirm that each variant is a real germline mutation present only in the breast cancer-affected members.

Through these processes, we identified 23 germline mutations in breast cancer-affected members in this family, of which 21 are novel mutations and 2 are rare mutations (rs143160739, rs370052455) with minor allele frequency (MAF) <0.001 (Table 1). The total frequency of the 23 mutations is 54, including 12 (52%) mutations present between 2 to 6 members, and 11 (48%) mutations present only in a single individual. On average, 9.2 mutations (54/6) are present in each breast cancer-affected member.

**Table 1:** Exome data and variant calls

**Case**	**Total bases**	**Coverage**	**Variant called**
1	5,333,230,980	133	46,317
2	3,715,176,060	93	43,510
3	8,558,235,540	213	50,533
4	5,621,290,920	140	47,064
5	3,915,557,820	98	45,531
6	3,209,882,580	80	40,598
7	3,605,428,440	90	40,211
8	4,143,350,070	103	43,996
Average	4,762,769,051	119	44,720

### Distribution of the mutations in the family

Of the 23 mutations, 12 were shared in at least two of the three *BRCA1*+ breast cancer-affected sisters (#1, #2, #4), 2 were present only in sister #2 (Table 2A). We compared the variants between mother and daughter pairs. Pair 1 includes mother (#2) and two daughters (#5, #6), all are *BRCA1*+ and affected with breast cancer at 53, 36 and 36 years old respectively; Pair 2 includes mother (#4) and daughter (#7), both are *BRCA1*+ and were affected with breast cancer at 35 years old. Because the variants shared between the father and the daughter(s) were eliminated, each mutation is firmly determined as either germline mutation inherited from the mother, or somatic mutation generated *de novo* in the daughter(s). Of the 23 mutations, 15 were germline mutations between the mother and the daughter(s) (9 in Pair 1, and 6 in Pair 2); 9 were *de novo* somatic mutation only in the daughter (#5). There was no *de novo* somatic mutation in the daughter (#6) in Pair 1 or the daughter (#7) in Pair 2 (Table 2B).

**Table 2:** Germline mutations identified in the family

**Gene**	**Position**	**Base change**	**AA change**	**Type**	**SIFT**		**PolyPhen 2**		**Frequency**
					**Score**	**Prediction**	**Score**	**Prediction**	
*SRA1*	chr5:139936828	c.C91T	p.P31S	SNV	0.00	D	1.00	D	6
PHKG2*	chr16:30767746	c.C706T	p.R236W	SNV	0.00	D	1.00	D	5
*ZNF24*	chr18:32919897	c.T464G	p.M155R	SNV	0.02	D	0.00	B	4
*TMPRSS7*	chr3:111797705	c.G1963A	p.G655S	SNV	0.00	D	1.00	D	4
ABLIM2	chr4:8055946	c.G791A	p.R264Q	SNV	0.05	D	1.00	D	3
*FOXP1*	chr3:71247489	c.C44T	p.A15V	SNV	0.34	T	0.98	D	3
*GSTK1*	chr7:142964824	c.G703A	p.G235R	SNV	0.02	D	1.00	D	3
*LACRT*	chr12:55028594	c.C32T	p.A11V	SNV	0.00	D	0.99	D	3
*PAMR1*	chr11:35456266	c.G1420A	p.G474R	SNV	0.00	D	1.00	D	3
*TTN*	chr2:179400887	c.G73392A	p.W24464X	Stop gain	NA	NA	NA	NA	3
*UEVLD*	chr11:18553971	c.G1312T	p.V438L	SNV	0.37	T	0.96	D	2
*ITGA1*	chr5:52240783	c.C3296G	p.S1099C	SNV	0.01	D	1.00	D	2
*BPTF*	chr17:65850386	c.A944C	p.N315T	SNV	0.00	D	1.00	D	1
*CACNB3*	chr12:49220218	c.G688A	p.A230T	SNV	0.01	D	1.00	D	1
*CUL4B*	chrX:119680410	c.A838T	p.R280X	Stop gain	NA	NA	NA	NA	1
*SEMA3C*	chr7:80374250	c.G2216A	p.R739Q	SNV	0.01	D	0.99	D	1
*TBC1D22B*	chr6:37280778	c.G1067A	p.S356N	SNV	0.03	D	0.39	B	1
*CHCHD1*	chr10:75541868	c.G35T	p.R12L	SNV	0.01	D	0.99	D	1
KCTD8*	chr4:44177010	c.C1219T	p.R407C	SNV	0.00	D	1.00	D	1
*CLCN4*	chrX:10176455	c.G1214A	p.C405Y	SNV	0.00	D	0.98	D	1
*LAT*	chr16:29000901	c.G634T	p.A212S	SNV	0.02	D	1.00	D	1
*ZNF304*	chr19:57868409	c.A1172T	p.Y391F	SNV	0.16	T	1.00	D	1
ZNF674	chrX:46359537	c.C1487A	p.P496H	SNV	0.00	D	1.00	D	1

*Two mutations exist in ESP 6500 with MAF 0.000077, all others are novel mutations only detected in this family.

D: probably damaging; P: possibly damaging; B: benign; T: tolerant.

### Chromosomal distribution and ratio of transition/transversion of the mutations

The 23 mutations are enriched in several chromosomes, such as chromosome 3 (*TMPRSS7, FOXP1*), chromosome 16 (*LAT, PHKG2*) and chromosome X (*CLCN4, CUL4B, ZNF674*). Except the mutations in *ZNF304* and *ZNF674*, no mutations are located in the repetitive sequences of SINE, LINE, LTR, simple or satellite sequences. Fifteen mutations are located in the regions with known structural variations and 5 mutations in the chromosomal fragile sites of 4A, 11E, 2G, 11C, and 18A (Table 3). For the 23 mutations, the ratio of transition/transversion (Ti/Tv) is 1.6. However, the ratio increased to 3.0 (9/3) for the common mutations. In contrast, the ratio decreased to 0.8 (5/6) for the mutations present only in single individual. The ratio between common mutations and individual mutations is statistically different by Fisher exact test (p = 0.009, Table 4).

**Table 3:** Mutation distribution in the family

**Gene**	**Position**	**Base change**	**AA change**	**Sister #1**	**Sister #2**	**Sister #4**	**Sister #3**
				**Cancer**	**Cancer**	**Cancer**	**Unaffected**
A. Mutation distribution in the three breast cancer-affected sisters							
*PHKG2*	chr16:30767746	c.C706T	p.R236W	+	+	+	-
*GSTK1*	chr7:142964824	c.G703A	p.G235R	+	+	-	-
*ITGA1*	chr5:52240783	c.C3296G	p.S1099C	+	+	-	-
*PAMR1*	chr11:35456266	c.G1420A	p.G474R	+	+	-	-
*TMPRSS7*	chr3:111797705	c.G1963A	p.G655S	+	+	-	-
*UEVLD*	chr11:18553971	c.G1312T	p.V438L	+	+	-	-
*LACRT*	chr12:55028594	c.C32T	p.A11V	+	-	+	-
*SRA1*	chr5:139936828	c.C91T	p.P31S	+	-	+	-
*ZNF24*	chr18:32919897	c.T464G	p.M155R	+	-	+	-
ABLIM2	chr4:8055946	c.G791A	p.R264Q	-	+	+	-
*FOXP1*	chr3:71247489	c.C44T	p.A15V	-	+	+	-
*TTN*	chr2:179400887	c.G73392A	p.W24464X	-	+	+	-
*CACNB3*	chr12:49220218	c.G688A	p.A230T	-	+	-	-
*CHCHD1*	chr10:75541868	c.G35T	p.R12L	-	+	-	-
B. Mutation distribution between generations							
Pair 1				Father #9	Mother #2	Daughter #5	Daughter #6
*RAB3C*	chr5:58147140	c.G646A	p.E216K	-	+	+	+
*SRA1*	chr5:139936828	c.C91T	p.P31S	-	+	+	+
*TMPRSS7*	chr3:111797705	c.G1963A	p.G655S	-	+	+	+
*ZNF24*	chr18:32919897	c.T464G	p.M155R	-	+	+	+
*LACRT*	chr12:55028594	c.C32T	p.A11V	-	+	+	-
*PAMR1*	chr11:35456266	c.G1420A	p.G474R	-	+	+	-
*GSTK1*	chr7:142964824	c.G703A	p.G235R	-	+	-	+
*PHKG2*	chr16:30767746	c.C706T	p.R236W	-	+	-	+
*UEVLD*	chr11:18553971	c.G1312T	p.V438L	-	+	-	+
*ITGA1*	chr5:52240783	c.C3296G	p.S1099C	-	+	-	-
*BPTF*	chr17:65850386	c.A944C	p.N315T	-	-	+	-
*TBC1D22B*	chr6:37280778	c.G1067A	p.S356N	-	-	+	-
*SEMA3C*	chr7:80374250	c.G2216A	p.R739Q	-	-	+	-
*CUL4B*	chrX:119680410	c.A838T	p.R280X	-	-	+	-
*CLCN4*	chrX:10176455	c.G1214A	p.C405Y	-	-	+	-
*LAT*	chr16:29000901	c.G634T	p.A212S	-	-	+	-
*ZNF304*	chr19:57868409	c.A1172T	p.Y391F	-	-	+	-
ZNF674	chrX:46359537	c.C1487A	p.P496H	-	-	+	-
*KCTD8*	chr4:44177010	c.C1219T	p.R407C	-	-	+	-
Pair 2				Father #10	Mother #4	Daughter #7	Son #8
*SRA1*	chr5:139936828	c.C91T	p.P31S	-	+	+	+
*PHKG2*	chr16:30767746	c.C706T	p.R236W	-	+	+	-
*CASQ1*	chr1:160165804	c.G769A	p.E257K	-	+	+	-
*ABLIM2*	chr4:8055946	c.G791A	p.R264Q	-	+	+	-
*TTN*	chr2:179400887	c.G73392A	p.W24464X	-	+	+	-
*FOXP1*	chr3:71247489	c.C44T	p.A15V	-	+	+	-

**Table 4:** Genomic features of the mutations

**A. Genome distribution of the mutations**						
**Gene**	**Position**	**Repetitive sequence**	**Structural variation**	**Fragile site**		
*BPTF*	chr17:65850386	-	+	-	-	-
*CACNB3*	chr12:49220218	-	+	-	-	-
*CHCHD1*	chr10:75541868	-	+	-	-	-
*FOXP1*	chr3:71247489	-	+	-	-	-
*GSTK1*	chr7:142964824	-	+	-	-	-
*ITGA1*	chr5:52240783	-	+	-	-	-
*LACRT*	chr12:55028594	-	+	-	-	-
*PHKG2*	chr16:30767746	-	+	-	-	-
*SRA1*	chr5:139936828	-	+	-	-	-
*CLCN4*	chrX:10176455	-	+	-	-	-
*LAT*	chr16:29000901	-	+	-	-	-
*KCTD8*	chr4:44177010	-	+	-	-	-
*ABLIM2*	chr4:8055946	-	+	4A	5149099	8732840
*PAMR1*	chr11:35456266	-	+	11E	31043424	36443424
*TTN*	chr2:179400887	-	+	2G	169791754	182991755
*UEVLD*	chr11:18553971	-	-	11C	16143424	21643424
*ZNF24*	chr18:32919897	-	-	18A	32746002	37246002
*CUL4B*	chrX:119680410	-	-	-	-	-
*SEMA3C*	chr7:80374250	-	-	-	-	-
*TBC1D22B*	chr6:37280778	-	-	-	-	-
*TMPRSS7*	chr3:111797705	-	-	-	-	-
*ZNF304*	chr19:57868409	+	-	-	-	-
ZNF674	chrX:46359537	+	-	-	-	-
**B. Transition/ transversion of the mutations**						
**Wild type**	**Mutation**	**Wild type**	**Mutation**			
Common mutations (> = 2)						
C	T	T	G			
C	T	G	T			
C	T	C	G			
C	T					
G	A					
G	A					
G	A					
G	A					
G	A					
Individual mutations						
C	T	A	T			
G	A	A	T			
G	A	A	C			
G	A	G	T			
G	A	G	T			
		C	A			
**C. Transition/Transversion ratio**						
**Class**	**Transition**	**Transversion**	**Ratio**			
Total mutation	14	9	1.6			
Common	9	3	3			
Individual	5	6	0.8			

### Functional categories of the mutated genes

Multiple mutations have deleterious effects on functinal importance and breast cancer-related genes (Table 5, Additional file 1: Table S1). Followings are examples of the mutation-affected genes:

**Table 5:** Function of mutation-damaged genes

**Genes**	**Function**	**Mutation type**
ABLIM2	Actin binding	nonsynonymous SNV
BPTF	Transcriptional regulation	nonsynonymous SNV
CACNB3	Calcium channel	nonsynonymous SNV
CHCHD1	Nuclear protein	nonsynonymous SNV
CLCN4	Chloride channel	nonsynonymous SNV
CUL4B	Polyubiquitination	stop gain
FOXP1	Transcriptional regulation	nonsynonymous SNV
GSTK1	Cellular detoxification	nonsynonymous SNV
ITGA1	Cell-cell adhesion	nonsynonymous SNV
KCTD8	Potassium channel	nonsynonymous SNV
LACRT	Lacrimal gland development	nonsynonymous SNV
LAT	TCR - and pre-TCR-mediated signaling	nonsynonymous SNV
PAMR1	Muscle regeneration	nonsynonymous SNV
PHKG2	Phosphorylase kinase for glycogenesis	nonsynonymous SNV
SEMA3C	Developmental regulation	nonsynonymous SNV
SRA1	Nuclear and non-nuclear receptor Regulation	nonsynonymous SNV
TBC1D22B	Unknown	nonsynonymous SNV
TMPRSS7	Serine protease for peptide hydrolyzes	nonsynonymous SNV
TTN	Structural protein for chromosomes	stop gain
UEVLD	Unknown	nonsynonymous SNV
ZNF24	Transcriptional regulation	nonsynonymous SNV
ZNF304	Transcriptional regulation	nonsynonymous SNV
ZNF674	Transcriptional regulation	nonsynonymous SNV

BPTF is a bromodomain PHD finger transcription factor, involved in transcriptional regulation and chromatin remodeling [28]. Copy number changes in BPTF are present in many types of cancer [29].

CUL4B is an E3 ubiquitin ligase catalysing polyubiquitination for protein degradation [30].

FOXP1 is a member of the forkhead box (FOX) transcription factor family, involving in regulation of tissue- and cell type-specific gene expression. Its expression is under the regulation of estrogen and it is known to play a role in breast cancer cell proliferation [31].

GSTK1 (glutathione S-transferase kappa 1) is involved in cellular detoxification [32].

LACRT (lacritin) is highly expressed in lacrimal glands. Copy number amplification of LACRT was observed in breast cancer [33].

PHKG2 is a phosphorylase kinase. It is involved in liver glycogenesis. Mutations in this gene cause glycogen storage disease type 9C [34].

SRA1 is a steroid receptor activator, involving in the regulation of many nuclear and non-nuclear receptors and associating with breast cancer [35]. The mutation in this gene is also present in the member (#8), who is a male not affected with breast cancer.

### Comparison between germline mutations and somatic mutations in breast cancer

The Cancer Genome Atlas Network (TCGA) identified 56 somatically mutated genes from 510 breast cancer tissues, of which about 90% are sporadic breast cancer [36]. Comparison between the 23 mutated genes in our study and the 56 mutated genes shows no overlap between the two sets of mutation-affected genes. The closest correlation is the mutations in the *FOX* family, in which *FOXA1* is somatically mutated in sporadic breast cancer and *FOXP1* is germline-mutated in the *BRCA1*+ family. Mutations in these two *FOX* genes are associated with breast cancer [37]. Absence of overlapping mutations between the *BRCA1*+ genomes and sporadic breast cancer genomes suggests the different genetic basis between these two types of breast cancer. Searching the 23 mutations by gene name in the COSMIC database, which contain somatic mutation information for various types of human cancer, shows nineteen genes being present in COSMIC, however the mutations were at different position; searching by exact positions shows the presence of the same mutations for *KCTD8* and *CACNB3. KCTD8* is a component of potassium channel and *CACNB3* is *a* subunit of calcium channel. The oncological roles of these two mutated genes in familial breast cancer remain to be elucidated.

## Discussion

Tumorigenesis requires multiple genetic defects to transform a normal cell to a tumor cell [38,39]. In familial breast cancer, germline mutations inherited from ancestors play important roles in cancer processes. *BRCA1*+ is the strongest germline predisposition for familial breast cancer. While it is widely accepted that *BRCA1*+ leads to breast cancer by causing genome instability, the detailed mechanism for how *BRCA1+* causes genome instability remain to be determined. Our study shows the presence of mutations in blood cells from the *BRCA1*+ breast cancer family. By definition, only the mutations occurred shortly after fertilization can be germline mutation. Although blood cells are frequently used in solid tumor genetic study to represent germline genome, the mutations detected in blood cells in fact include both true germline mutations occurred during fertilization and somatic mutations occurred after fertilization. By combining the pedigree information, however, we can clearly distinguish the two types of mutations that those shared between generations (mothers and daughters) are the germline mutation, and those only presented in single individual (#2 daughter) are *de novo* somatic mutations. The number of germline mutations is much larger than the number of *de novo* somatic mutations, this is due to the fact that germline mutations are accumulated/inherited from multiple generations whereas somatic mutations are only present in individual generation. The results indicate that *BRCA1+* can induce germline genome instability represented by the genome of blood cells.

Can the small numbers of mutations be identified by chance? For the following reasons, we consider it unlikely: in the study, we applied a multi-step mapping pipeline in order to maximally differentiate germline mutations associated with cancer from abundant normal variants, including the filtration of normal variants using public variant databases of dbSNP, 1000 genomes and exome variation databases, the use of the aged, unaffected family members to remove private variants in the family, exclusion of the contribution of father’s germline mutation to the daughter in the trios, the focus only on the deleterious mutations causing non-synonymous mutations, splicing alternation and stop gain/loss, and the use of Sanger sequencing validation. It is also worth indicating that the functionally important mutations in cancer are in small numbers [40,41]. The small number of mutations identified were likely generated by *BRCA1*+ directly or indirectly. Their roles could be promoting oncogenesis, or function as *BRCA1*+ modifiers to amplify the oncogenic function of *BRCA1*+. Our study focuses on the deleterious mutations at single-based level. Other types of genetic changes could also be present in BRCA1+ genome. For example, increased CNV in TP53 was shown to be present in Li-Fraumeni syndrome, a disease closely related with BRCA1+ familial breast cancer [42].

It is well known that transition occurs at a higher rate than transversion mutations in cancer cells [40]. Indeed, we observed a higher Ti/Tv ratio (3.0) than that in the normal genome (2.1) for the common mutations, implying that the germline genome instability also follows the same trend that found in somatic genome mutation. However, the ratio of Ti/Tv mutations in the individual mutations (0.8) is lower than that in the normal genome. Possible causes could be that the individual mutations tend to be random events, with less biological significance, or that the number of those mutations is too small for the comparison.

Elimination of family-specific normal variants is a key to identify the true predispositive mutations in the cancer, as a normal human genome can have multiple genes mutated [43]. Familial-specific normal variants cannot be removed solely by referring to the population-based variation databases, as many familial-specific normal variants are not included. The unaffected family members can serve as the closest control for this purpose. While this process could remove certain real mutations shared between the unaffected and affected members (low penentrant), and certain family-specific normal variants could still remain in the affected members (not present in the unaffected members), the mutations present only in the affected members are more likely to be associated with cancer than those shared between the affected and unaffected members. Referring to functional importance of the mutation-affected genes also helps to identify ture predispositive mutations. Because of the mutations we identified are present only in the cancer members, shared between multiple cancer-affected members in the disease family, and have functional relevance to cancer, they are most likely associated with breast cancer in the disease family.

## Conclusions

Our study shows the presence of genome instability in the genomes of non-breast cells in the *BRCA1*+ familial breast cancer family. The presence of germline mutations provides a potential source to identify genetic targets for early intervention of tumorogenesis process in *BRCA1*+ carriers long before tumor formation in breast cells, for which there are currently limited options besides preventive surgery.

### Availability of supporting data

The exome data generated by the sutdy were deposited in NCBI (Accession number SRP028652). url: http://www.ncbi.nlm.nih.gov/Traces/sra/?study=SRP028652

## Competing interests

The authors declare that they have no competing interests.

## Authors’ contributions

FX, HW, PXC, BD performed experiments, YK performed bioinformatic data analysis, CS, DB identified the subjects and prepared DNA samples, JL performed statistical analysis, KY. HL, SMW conceived the study, SMW designed the experiment and wrote the paper. All authors read and approved the final manuscript.

## Pre-publication history

The pre-publication history for this paper can be accessed here:

http://www.biomedcentral.com/1471-2407/14/342/prepub

## Supplementary Material

Additional file 1: Table S1 Validation by Sanger sequencing. Click here for file
